# Platelet and ferritin as early predictive factors for the development of macrophage activation syndrome in children with Kawasaki disease: A retrospective case-control study

**DOI:** 10.3389/fped.2023.1088525

**Published:** 2023-02-15

**Authors:** Hua-yong Zhang, Min Xiao, Dan Zhou, Fan Yan, Yong Zhang

**Affiliations:** ^1^ Department of Cardiology, Wuhan Children’s Hospital/Wuhan Maternal and Child Healthcare Hospital, Tongji Medical College, Huazhong University of Science & Technology, Wuhan, China; ^2^ Department of Rheumatology, Wuhan Children’s Hospital/Wuhan Maternal and Child Healthcare Hospital, Tongji Medical College, Huazhong University of Science & Technology, Wuhan, China; ^3^ Department of Critical Care Medicine, Wuhan Children’s Hospital/Wuhan Maternal and Child Healthcare Hospital, Tongji Medical College, Huazhong University of Science & Technology, Wuhan, China

**Keywords:** kawasaki disease, macrophage activation syndrome, early predictive factor, children, retrospective case-control study

## Abstract

**Objective:**

To investigate the early predictive factors for Kawasaki disease complicated with macrophage activation syndrome (KD-MAS)

**Methods:**

We performed a retrospective case-control study in children with KD from August 2017 to August 2022, involving 28 cases with KD-MAS and 112 cases not developing KD-MAS. Based on the univariate analysis, binary logistic regression was used to identify the early predictive factors for KD-MAS development, and the receiver operating characteristic curve (ROC) analysis was carried out to obtain the optimal cut-off value.

**Results:**

Two predictive factors were associated with the development of KD-MAS, which were PLT (*OR *= 1.013, 95%*CI*, 1.001–1.026), and serum ferritin (*OR *= 0.991, 95%*CI*, 0.982–0.999). The cut-off value of PLT was 110 × 10^9^/L, and the cut-off value of serum ferritin was 548.4 ng/ml.

**Conclusion:**

Children with KD who had a PLT count under 110 × 10^9^/L, and a serum ferritin level over 548.4 ng/ml are more likely to develop KD-MAS.

## Introduction

Kawasaki disease (KD) is an idiopathic autoimmune vasculitis, which is most frequently observed in children younger than 5 years of age (1). During the acute phase of KD, the aggravation of the condition can result in macrophage activation syndrome (MAS), which is a rare and serious complication of KD and is also known as secondary hemophagocytic lymphohistiocytosis (HLH) associated with rheumatic diseases (2). Two forms of HLH can be distinguished, primary or familial HLH and secondary HLH. Primary HLH is caused by mutations in genes such as PFR1, STX11, and STXBP2, which encode perforin or proteins involved in cytotoxic lymphocyte degranulation (3). Secondary HLH can be triggered by infections, including Epstein-Barr viruses and adenoviruses (4, 5). Rheumatic diseases can also induce secondary HLH, including systemic juvenile idiopathic arthritis (sJIA) and KD (2, 6, 7). There is less clarity in separating primary HLH from secondary HLH since primary HLH can be caused by secondary factors, whereas secondary HLH may also be caused by genetic mutations (8, 9). The pathogenesis of MAS is not completely understood, some scholars believe that MAS is an inflammatory condition caused by excessive activation of macrophages and T cells (10).

Some children have been reported to develop Multisystem Inflammatory Syndrome (MIS-C) after exposure to SARS-CoV-2 in the Era of COVID-19. The syndrome of MIS-C mimics KD, which has also been described as SARS-CoV-2-induced Kawasaki-like syndrome (11). The onset of MIS-C occurs approximately 4 weeks after symptomatic or asymptomatic SARS-CoV-2 infection (12). If MIS-C is refractory to treatment, the development of MAS should be suspect, which can be fatal if not diagnosed early (13). The study has reported that higher procalcitonin (PCT), ferritin, and fibrinogen (FIB) levels at admission were the risk factors for MAS in MIS-C. Fortunately, the clinical courses and prognoses of MAS in MIS-C seem better than other rheumatological diseases based on limited data (13). However, multi-center long-term observations are needed to determine its truth.

Recently, an increased incidence of KD complicated with MAS (KD-MAS) has been observed, ranging from 1.1% to 1.9%. However, the incidence may have been underestimated due to the strict diagnostic criteria (2, 6, 14). The early symptoms of KD-MAS are not typical, and it is difficult to differentiate from KD-MAS to intravenous immunoglobulin (IVIG) resistant KD. Delay in diagnosis and treatment leads to a high fatality ratio (2, 15). Therefore, early recognition and treatment appear important in patients with KD-MAS. According to previous studies, KD-MAS is characterized by prolonged fever, splenomegaly, liver dysfunction, hypofibrinogenemia, hyperferritinemia, hypertriglyceridemia, and pancytopenia (2, 6, 14). However, it is lacking more specific indicators and precise quantitative studies. Consequently, this study retrospectively aims to explore the early predictive factors of KD-MAS and provides quantitative evidence of the early-warning signals.

## Materials and methods

### Study design and participants

In this retrospective case-control study, all patients with KD were enrolled between August 2017 and August 2022 at Wuhan Children's Hospital. The diagnosis criteria for KD were based on the guideline issued by the Japan Kawasaki Disease Research Committee in 2020 (16). MAS diagnosis was made according to the MAS-sJIA-2016 criteria (17).

Inclusion criteria: (1) individuals younger than 18 years (2); complete or incomplete KD was diagnosed (3); MAS was diagnosed based on the diagnostic criteria. Exclusion criteria: (1) individuals without complete medical records (2); hospitalization for less than 24 h and referral to other hospitals (3); treatment with IVIG or steroids in other hospitals before admission.

Individuals were divided into two groups based on their clinical outcome: KD and KD-MAS groups. In total, 28 cases satisfied the KD-MAS criteria. In the KD group, 4 control cases were chosen for each patient and matched to its control by admission time (±1 week) as the matching factor to control for the effect of seasonal factor (18, 19). This study was approved by the Ethics Committee of Wuhan Children's Hospital.

Our primary objective was to investigate the early predictor factors for KD-MAS, and the secondary objective was to evaluate the evolution of KD-MAS during medical treatment. IVIG resistance, as a potential predictor factor, was defined as persistent or recrudescent fever at least 36 h and <7 days after the completion of the first IVIG infusion (1).

### Data collection

Based on the electronic medical records of individuals enrolled in this study, the following clinical and laboratory data were reviewed: (1) general demographic data: age at admission, sex; (2) clinical manifestations: length of fever duration before admission, length of illness at primary IVIG treatment, incidence of splenomegaly, incomplete KD, IVIG-resistance KD, coronary artery lesions, and mortality; (3) laboratory indicators: white blood cell count (WBC), neutrophils count, neutrophil-to-lymphocyte count ratio (NLR), platelet (PLT), hemoglobin (Hb), hypersensitive C-reactive protein (Hs-CRP), erythrocyte sedimentation (ESR), PCT, lactic dehydrogenase (LDH), aspartate aminotransferase (AST), alanine aminotransferase (ALT), serum electrolytes, serum ferritin, coagulation function, serum lipids, inflammatory cytokines, and other tests. The assessment of laboratory data was collected based on the worst value indicators during the acute period of KD and before the first dose of IVIG therapy. For the individuals of KD-MAS, the laboratory indicators 36–72 h after the first dose of IVIG treatment and at the time of KD-MAS diagnosis were collected for further analysis.

### Statistical analysis

IBM SPSS (version 22.0, Armonk, NY, United States) software and R version 4.1.3 were used for the statistical analysis. Counting data were expressed as the number of cases and percentage [n (%)], and were evaluated using Chi-square (*χ*^2^) tests or Fisher's exact tests. the normal distribution data are expressed as means ± standard deviation $\left(\bar{x}\pm s\right)$, and the differences between groups were compared using independent-sample *t*-tests. For the non-normal distribution data, expressed as a median and interquartile range [M (*P_25_, P_75_*)], non-parametrical tests, such as the Mann–Whitney *U* test was used. The early predictive factors for KD-MAS development were evaluated using binary logistic regression analyses. The prognostic value of predictive factors was evaluated using receiver operator characteristic curves (ROC) and areas under the ROC curves (AUC). KD-MAS is a rare disease and the number of cases is relatively small, we faced a typical prediction modeling problem with small sample sizes. The high number of variables included in the logistic regression models may have resulted in an additional risk of overfitted effect. Therefore, Leave-one-out cross-validation was used to evaluate the performance of the logistic regression model. A model with an AUC over 0.7 was considered to be of clinical value (20).

## Results

### Baseline characteristics

**Figure 1:**
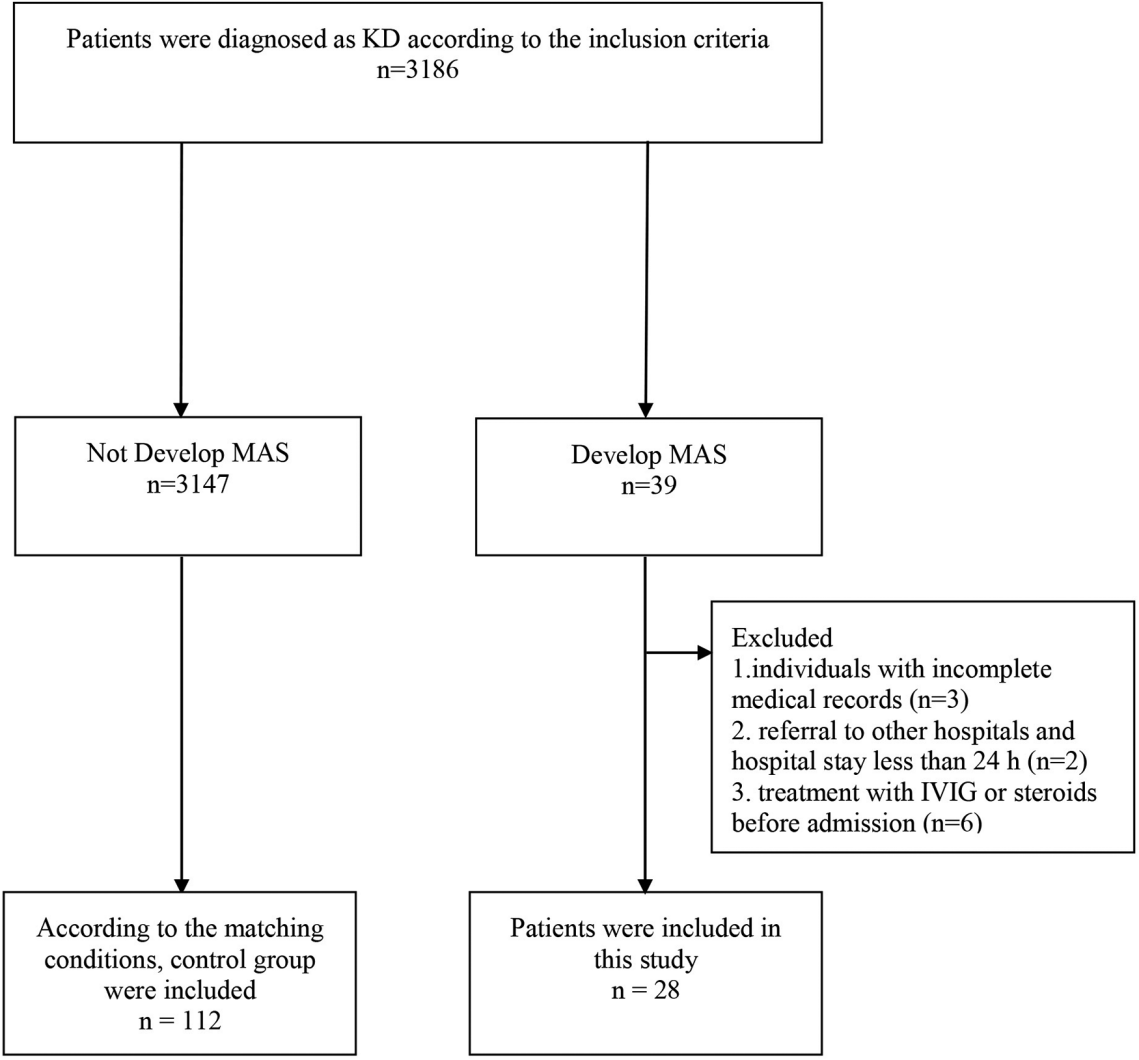
the flow diagram of patients included in the study analysis.

From August 2017 to August 2022, a total of 3186 cases were admitted with a discharge diagnosis of KD. 39 (1.2%) of these patients were diagnosed with KD-MAS during hospitalization. Of these patients, 11 patients were excluded, including 3 cases without complete medical records, 2 cases transferring to other hospitals and hospital stay less than 24 h, and 6 cases of treatment with IVIG or steroids in other hospitals before admission. 28 KD-MAS patients met the inclusion criteria and were enrolled in this study. Over the same period, 112 KD cases were enrolled in this study as the control group. The recruiting procedure is described in Figure 1. Meanwhile, the baseline clinical features and outcomes of patients between the two groups are presented in Table 1.

**Table 1:** Comparison of the clinical characteristics and outcomes.

Characteristic	KD (*n* = 112)	KD-related MAS (*n* = 28)	*χ^2^/Z/t*-test	*p*
Demographics				
Male (*n*, %)	68(60.7)	19(67.8)	0.486	0.486
Age [years, M (*P_25_, P_75_*)]	1.87 (1.00,3.33)	2.24 (1.39,4.42)	−1.402	0.161
<1 year (*n*, %)	26(23.2)	4(14.3)	1.061	0.303
1–5 years (*n*, %)	78 (69.6)	19 (67.8)	0.034	0.855
≥5 years (*n*, %)	8(7.1)	5(17.9)	3.053	0.081
Clinical characteristics				
Length of fever duration before admission (d, $\bar{x}\pm s$)	5.27 ± 1.78	5.71 ± 2.45	1.097	0.274
Incomplete KD (*n*, %)	21 (18.8)	5 (17.9)	0.012	0.913
IVIG-resistance KD (*n*, %)	13(11.6)	25 (89.3)	68.348	0.000
Splenomegaly (*n*, %)	11(9.8)	23(82.1)	63.717	0.000
Length of illness at primary IVIG treatment (d, $\bar{x}\pm s$)	6.05 ± 1.55	6.46 ± 1.95	−1.073	0.283
≤4 days (*n*, %)	7(6.3)	4 (14.3)	1.998	0.158
≤5 days (*n*, %)	39(34.8)	8(28.6)	0.392	0.531
Laboratory findings				
WBC (×10^9^/L, $\bar{x}\pm s$)	12.29 ± 4.75	11.91 ± 8.15	−0.326	0.745
Neutrophils count (×10^9^/L, $\bar{x}\pm s$)	8.14 ± 3.82	7.62 ± 5.58	−0.586	0.559
NLR [M (*P_25_, P_75_*)]	7.68(5.35,10.30)	7.19(2.67,11.10)	−0.328	0.743
PLT [×10^9^/L, M (*P_25_, P_75_*)]	343.00 (262.50,447.75)	69.00 (48.50,177.25)	−6.382	0.000
Hb (g/L, $\bar{x}\pm s$)	105.89 ± 8.53	102.00 ± 10.97	−1.722	0.087
ESR [mm/H, M (*P_25_, P_75_*)]	51.00 (34.50,74.00)	47.50 (38.75,72.75)	−0.589	0.556
Hs-CRP [mg/L, M (*P_25_, P_75_*)]	80.45(46.85,117.00)	105.50(61.00,156.00)	−1.810	0.070
PCT [mg/L, M (*P_25_, P_75_*)]	1.17 (0.40,2.25)	1.54 (0.58,3.08)	−1.430	0.153
CL^−^ [mmol/L, M (*P_25_, P_75_*)]	99.65 (97.40,101.68)	99.35 (96.85,100.90)	−.0.899	0.369
Na^+^ [mmol/L, M (*P_25_, P_75_*)]	136.60 (134.75,138.28)	136.25 (133.52,139.15)	−0.826	0.409
TG [mmol/L, M (*P_25_, P_75_*)]	1.26 (1.03,1.65)	2.43 (1.79,3.58)	−5.403	0.000
Ferritin [ng/ml, M (*P_25_, P_75_*)]	139.13(109.90,218.75)	935.66(655.50,2035.85)	−7.715	0.000
FIB [g/L, M (*P_25_, P_75_*)]	5.78(4.78,6.86)	1.65(133.53,139.15)	−7.380	0.000
Albumin [g/L, M (*P_25_, P_75_*)]	37.60 (34.73,40.35)	28.90 (24.15,35.48)	−4.931	0.000
ALT [U/L, M (*P_25_, P_75_*)]	26.50 (13.00,65.00)	94.50 (51.25,130.50)	−4.838	0.000
AST [U/L, M (*P_25_, P_75_*)]	31.00 (22.00,43.75)	78.00 (47.00,140.25)	−4.565	0.000
LDH [U/L, M (*P_25_, P_75_*)]	273.00 (239.00,338.75)	450.50 (336.50,847.00)	−5.444	0.000
IL-2 [pg/ml, M (*P_25_, P_75_*)]	3.56 (2.13,6.72)	3.56 (2.34,5.49)	−0.240	0.811
IL-4 [pg/ml, M (*P_25_, P_75_*)]	3.63 (2.56,6.44)	3.78 (2.78,5.93)	−0.086	0.931
IL-6 [pg/ml, M (*P_25_, P_75_*)]	113.95 (44.45,181.19)	123.81 (75.60,225.60)	−1.159	0.246
IL-10 [pg/ml, M (*P_25_, P_75_*)]	13.12 (7.47,21.26)	23.04 (15.08,30.16)	−2.873	0.004
TNF-α [pg/ml, M (*P_25_, P_75_*)]	4.82 (3.30,6.77)	5.52 (2.76,11.44)	−1.055	0.291
INF-*γ* [pg/ml, M (*P_25_, P_75_*)]	7.05 (3.84,12.55)	6.88 (2.81,14.88)	−0.291	0.835
Outcomes				
Coronary artery lesions (*n*, %)	10(8.9)	8(28.6)	7.714	0.005
Mortality (*n*, %)	1(0.9)	3(10.7)	7.785	0.005

KD, Kawasaki disease; MAS, macrophage activation syndrome; IVIG, intravenous immunoglobulin; NLR, neutrophil-to-lymphocyte count ratio; WBC, white blood cell count; PLT, platelet; Hb, hemoglobin; Hs-CRP, hypersensitive C-reactive protein; ESR, erythrocyte sedimentation; PCT, procalcitonin; TG, triglyceride; FIB, Fibrinogen; ALT, alanine aminotransferase; AST, aspartate aminotransferase; LDH, lactic dehydrogenase; IL, interleukin; TNF-α, tumor necrosis factor α; INF-γ, interferon γ.

In the KD-MAS group, 23 (82.1%) cases could also fulfill the HLH-2004 criteria. According to bone marrow examinations, 14 (50.0%) cases displayed macrophage proliferation and hemophagocytosis. 13 (46.4%) cases presented with NK cell defects, including reduced NK cell numbers, and reduced NK cell activity. Laboratory indicators showed that PLT count, triglyceride (TG), and serum ferritin level increased significantly (*p *< 0.05), whereas Hs-CRP, PCT, and Hb level decreased (*p *< 0.05), 36–72 h after the first dose of IVIG treatment. Additionally, the other laboratory findings of KD-MAS patients before and after the first dose of IVIG treatment are listed in the Supplementary Table. Multisystemic complications of varying degrees were found in all cases. 22 (78.6%) cases had hypoalbuminemia, 16 (57.1%) cases had liver function damage, 9 (36.0%) cases had acute heart failure, 8 (28.6%) cases had coronary artery lesions (CALs), 6 (21.4%) cases had aseptic meningitis, 6 (21.4%) cases had KD shock syndrome (KDSS), 5 (17.9%) cases had acute kidney impairment, 3 (10.7%) cases had incomplete intestinal obstruction, and 3 (10.7%) cases had disseminated intravascular coagulation and multiple organ failure. Genetic analysis of familial HLH was performed in 5 (17.9%) patients with KD-MAS, and STXBP2 gene mutation was identified in an 8 years old girl. 15 (53.6%) cases received one dose of IVIG combined with glucocorticoid therapy, and 13 (46.4%) cases received a second dose of IVIG (2 g/kg, twice) combined with glucocorticoid therapy. After discussion, cyclosporine A (CsA) was added in 6 patients for complementary therapy, and 4 (14.3%) cases received chemotherapy according to the 2004-HLH protocol. At discharge, the clinical outcome of 25 (89.3%) cases was favorable, and 3 (10.7%) cases died of disseminated intravascular coagulation and multiple organ failure. During the follow-up period, 2 (7.1%) cases had recurrent KD events.

### Early predictive factors for patients with KD to develop MAS

To determine the relative effect of each factor for KD-MAS, we conducted a logistic regression analysis. The univariate analysis identified splenomegaly (*OR *= 0.024, 95%*CI*, 0.007–0.075, *p* =0.000), IVIG-resistance KD (*OR *= 0.016, 95%*CI*, 0.004–0.060, *p* =0.000), PLT (*OR *= 1.015, 95%*CI*, 1.010–1.020, *p* =0.000), AST (*OR *= 0.986, 95%*CI*, 0.978–0.994, *p* =0.001), ALT (*OR *= 0.990, 95%*CI*, 0.984–0.996, *p* =0.001), LDH (*OR *= 0.993, 95%*CI*, 0.989–0.996, *p* =0.000), TG (*OR *= 0.190, 95%*CI*, 0.097–0.370, *p* =0.000), FIB (*OR *= 4.872, 95%*CI*, 2.776–8.551, *p* =0.000), serum ferritin (*OR *= 0.991, 95%*CI*, 0.987–0.994, *p* =0.000), and serum albumin (*OR *= 1.271, 95%*CI*, 1.159–1.393, *p* =0.000) as potential early predictive factors for KD to develop MAS. Multivariate analysis indicated that PLT (*OR *= 1.013, 95%*CI*, 1.001–1.026, *p* =0.048), and serum ferritin (*OR *= 0.991, 95%*CI*, 0.982–0.999, *p* =0.032) were independent early predictive factors for KD developing MAS (Table 2).

**Table 2:** Logistic regression analysis of early predictive factors for KD to develop MAS.

Factors	Univariate analysis		Multivariate analysis	
	*p*	*OR* (95% *CI*)	*p*	*OR* (95% *CI)*
Splenomegaly	0.000	0.024 (0.007, 0.075)	0.779	0.393 (0.001, 271.224)
IVIG-resistance KD	0.000	0.016 (0.004, 0.060)	0.599	0.121 (0.00, 317.001)
PLT	0.000	1.015 (1.010, 1.020)	0.048	1.013 (1.001, 1.026)
AST	0.001	0.986 (0.978, 0.994)	0.769	1.007 (0.964, 1.051)
ALT	0.001	0.990 (0.984,0.996)	0.745	0.993 (0.950, 1.038)
LDH	0.000	0.993 (0.989, 0.996)	0.448	0.992 (0.972, 1.013)
TG	0.000	0.190 (0.097, 0.370)	0.220	0.296 (0.042, 2.067)
FIB	0.000	4.872 (2.776, 8.551)	0.790	0.906 (0.440, 1.866)
ferritin	0.000	0.991 (0.987, 0.994)	0.032	0.991 (0.982, 0.999)
Albumin	0.000	1.271 (1.159, 1.393)	0.881	0.974 (0.695, 1.367)

KD, Kawasaki disease; MAS, macrophage activation syndrome; IVIG, intravenous immunoglobulin; PLT, platelet; AST, aspartate aminotransferase; ALT, alanine aminotransferase; LDH, lactic dehydrogenase; TG, triglyceride; FIB, Fibrinoge.

### The value of early predictive factors for patients with KD to develop MAS

**Figure 2:**
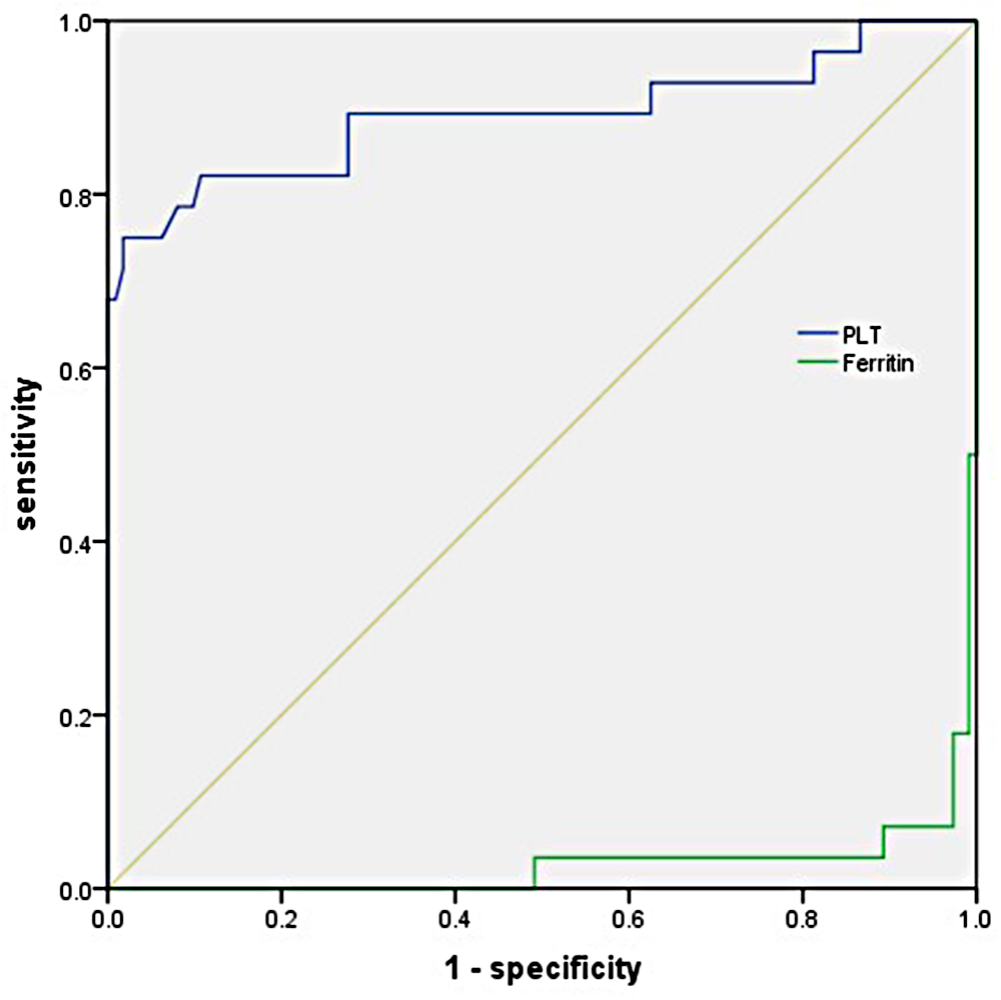
Receiver operating characteristic (ROC) curve analysis of PLT and serum ferritin in predicting the development of mas in children with KD.

Multivariate logistic regression analysis showed that PLT and serum ferritin were significantly associated with KD-MAS. To further examine the predictive value of the above factors, the ROC curve analysis was performed. The AUC of PLT was 0.891 (95%*CI*, 0.800–0.981, *p *< 0.001) for the prediction of KD-MAS, and the optimal cut-off point of PLT was 110 × 10^9^/L, with a sensitivity of 75.0% and a specificity of 98.2%. The AUC of serum ferritin was 0.972 (95%*CI*, 0.945–0.999, *p *< 0.001), and the optimal cut-off point was 548.4 ng/ml, with a sensitivity of 92.9% and a specificity of 97.3% (Figure 2). Based on this final model, the index of discrimination (AUC) equals 0.952 (0.905–0.999) (Supplementary Figure S1). Application of a leave-one-out cross-validation resulted in an AUC equal to 0.831 (0.735–0.926) (Supplementary Figure S2).

## Discussion

To the best of our knowledge, this study was the first to explore the early predictive factors for KD-MAS from a quantitative perspective. The present study found that two factors were associated with the development of KD-MAS, which were PLT (*OR *= 1.013, 95%*CI*, 1.001–1.026), and serum ferritin (*OR *= 0.991, 95%*CI*, 0.982–0.999). In prior qualitative studies, KD patients with persistent fever, male sex, older than 5 years of age, increased serum ferritin level, and hepatosplenomegaly tended to develop KD-MAS (2, 15). Interestingly, a similar phenomenon was also found in our present study, and the following findings can indicate KD tends to develop MAS: (1) splenomegaly, decreased PLT count, hypoproteinemia, hypofibrinogenemia, hyperlipidemia, hyperferritinemia, and elevation of transaminases and LDH levels on admission; (2) a significant increase in serum ferritin and TG levels, and a significant decrease in Hb level before and 36–72 h after the first dose of IVIG treatment (3); IVIG-resistance KD.

Serum ferritin is an important diagnostic marker in MAS, as it is in both the HLH-2004 criteria and the MAS-sJIA-2016 criteria. According to the HLH-2004 criteria, ferritin level is measured at 500 ng/ml, as compared to the MAS-sJIA-2016 criteria, at 684 ng/ml (7, 17). In KD-MAS, serum ferritin level was higher than in control KD patients at admission, which became significantly elevated as the disease progressed. When the diagnosis was confirmed, serum ferritin level was already significantly higher than the HLH-2004 criteria and the MAS-sJIA-2016 criteria. In this quantitative study, we have demonstrated that children with KD who had a serum ferritin level over 548.4 ng/ml before the first dose of IVIG treatment are more likely to develop KD-MAS.

PLT count is considered a biomarker for the development of CALs in KD, which usually reaches the lowest count within 6–7 days, and peaks after 10 days (21). KD patients with a PLT count under 150 × 10^9^/L or over 450 × 10^9^/L are at higher risk for CALs (21). In MAS, PLT is also an important diagnostic marker. A low platelet count is often the first laboratory finding in MAS (6). The cutoff value of thrombocytopenia is 100 × 10^9^/L by the HLH-2004 criteria, which is 181 × 10^9^/L by the MAS-sJIA-2016 criteria (7, 17). In the present study, KD patients with a PLT count under 110 × 10^9^/L before the first dose of IVIG treatment are more likely to develop KD-MAS.

As MAS was first reported in patients with sJIA, it was previously considered a form of secondary HLH associated with rheumatic diseases (6). KD-MAS is estimated to occur in 1.1–1.9% of KD patients, while sJIA patients are estimated to have a 10% incidence of MAS (2, 6, 7, 14). However, it has been questioned whether the HLH-2004 criteria and the MAS-sJIA-2016 criteria are too strict to diagnose KD-MAS in an early stage (6, 14). According to previous reports, only 74%–76% of KD-MAS patients fulfilled the HLH-2004 criteria or the MAS-sJIA-2016 criteria, and over 90% of cases were not diagnosed at an early stage, resulting in a poor prognosis with a mortality rate of 13%–25% (2, 6, 22–24). The reasons for this could be twofold. On the one hand, KD-MAS may be under-recognized due to a limited understanding of the diagnostic criteria, and on the other hand, not recognizing KD-MAS early may result in delayed definitive referral and treatment, contributing to disease progression and multisystemic complications (6, 14, 25). In our experience, a similar phenomenon indeed existed in our center. Earlier, we assessed the KD-MAS strictly according to the HLH-2004 criteria, resulting in a missed diagnosis or delayed diagnosis at the early stage. We only administered a second dose of IVIG for patients tending to develop KD-MAS as the therapy for IVIG-resistance KD instead of the earlier treatment with IVIG plus steroid therapy. This conservative treatment protocol could lead to more serious complications and even death. Later, we had to take more aggressive diagnostic and therapeutic strategies with accumulated experience, and complication rates have fallen appreciably, without death. Taken as a whole, the case fatality (10.7%) of KD-MAS in our study was low compared with reported rates in the literature (2, 6, 22). To date, almost all KD-MAS patients have required additional therapy after failing to respond to the first dose of IVIG, including a second dose of IVIG, glucocorticoid, CsA, methotrexate, etoposide, anakinra, infliximab, plasmapheresis, and HLH-2004 protocol (2, 6, 14, 17, 26). However, current studies about therapeutic approaches are limited and unsatisfactory. We do not know which additional therapy is more effective. Relevant studies have reported the HLH-2004 protocol was used in 24 KD-MAS patients, 7 of which died with a mortality rate of 29% (2). Therefore, careful attention should be paid to designing the treatment protocol. Based on our experience, earlier identification of progression means early and more effective treatment. Accordingly, we suggest that the second dose of IVIG combined with steroid therapy or steroid therapy alone for patients tending to develop KD-MAS should be implemented as early as possible in the acute phase. Besides this, the addition of monoclonal antibodies, CsA, or plasmapheresis is decided according to the clinical judgment of disease activity.

The pathogenesis of KD-MAS has not yet been clarified. An immune disorder characterized by uncontrolled T lymphocyte and macrophage activation and excessive production of inflammatory cytokines may participate in KD-MAS (2, 27). In previous studies, heterozygous mutations in primary HLH genes were found in as many as 40% of MAS patients, which induced the dysfunction of the perforin-mediated cytolytic pathway used by NK cells and cytotoxic CD8 T lymphocytes (9, 28, 29). In addition, mutations in a variety of other pathogenic pathways have been noted to lead to a similar cytokine storm syndrome in MAS, including SH2P1A, NLRC4, IKBKG, and LIPA genes (29). The whole-exome and whole-genome sequencing will likely identify novel MAS gene associations and noncoding mutations altering gene expression (9, 29). In the present study, genetic analysis of familial HLH was performed in 5 (17.9%) patients with KD-MAS, and STXBP2 gene mutation was identified in an 8 years old girl. This girl was clinically cured by chemotherapy therapy according to the HLH-2004 protocol. However, recurrence was observed after 2 years of follow-up. This reminds us that more attention should be paid to the investigation of the genetic background of KD-MAS.

Since the outbreak of COVID-19 infections in December 2019, the SARS-CoV-2-related MIS-C has been reported by several centers (11–13, 30, 31). The overall manifestations of MIS-C are similar to those of KD, including fever, conjunctivitis, and elevated inflammatory markers. Some children with MIS-C also met the criteria of KD (30). Therefore, it is important to explore the differences between MIS-C and KD. Studies reported that a higher frequency of gastrointestinal symptoms, shock, and reduced lymphocytes and macrophages is observed in MIS-C than in KD. Moreover, the MIS-C cases were significantly older than the KD patients, with a less rash rate and a higher ICU admission rate (11, 13, 30). The current treatment of MIS-C mimics the treatment of KD, and the use of IVIG and/or high-dose corticosteroids was recommended as first-line therapy in these patients (11, 32). Nonetheless, the optimal treatment regimen for MIS-C is uncertain. Both MIS-C and KD can also develop MAS as the disease progressed. To date, however, it is not clear whether MAS related to MIS-C and MAS related to KD share the same pathogenesis (11, 13, 33). The early differential diagnosis of sJIA, MIS-C, and KD is difficult. All the diagnoses of these diseases are made clinically, and Kawasaki-like or MIS-C-like phenotypes can be found in the early stage of sJIA. MAS is also an overlapping syndrome among KD, MIS-C, and sJIA (29, 34–36). There have been reported that 0.2% of KD patients were treated for KD and were eventually diagnosed with sJIA within 3–6 months after onset (34). Typically, KD is diagnosed before the 10th day of fever. If there is arthritis in 1 or more joints for at least 3 days or persistent fever for more than 2 weeks with at least 1 other accompanying sign of sJIA in KD patients, the diagnosis of sJIA should be considered (34). Those patients suspected to have KD but subsequently diagnosed with SJIA had a higher incidence of MAS than sole KD. Therefore, differential diagnosis and timely evaluation should be given more attention (34).

## Study limitations

There are several limitations to the present study. First, the retrospective design is susceptible to selection bias. Second, KD-MAS is a rare disease, and the total number of patients from the involved studies was relatively small, which does not satisfy the rule of the events per variable by logistics regression. The current results may be still not robust enough. However, considering that the results are somewhat interpretable, it is still presented. The reliability of this model needs to be confirmed by further studies. Third, some clinical missing indicators were not included in the analysis. Fourth, only a few poor prognosis cases were tested for familial HLH-associated genes, and the missed diagnoses could not be ruled out. Last, the study was a single-center retrospective study, the differences in patient selection and management strategies may limit the reproducibility. Thus, we need more multicenter studies to identify our findings.

## Conclusions

In conclusion, children with KD who had a PLT count under 110 × 10^9^/L, and a serum ferritin level over 548.4 ng/ml before the first dose of IVIG treatment are more likely to develop KD-MAS.

## Data Availability

'The original contributions presented in the study are included in the article/**Supplementary Materials**, further inquiries can be directed to the corresponding author/s.
