# Supplementary material for: Platelet and ferritin as early predictive factors for the development of macrophage activation syndrome in children with Kawasaki disease: A retrospective case-control study
Source: Front Pediatr. 2023 Feb 15;11:1088525. doi: 10.3389/fped.2023.1088525 (PMC9977190; doi:10.3389/fped.2023.1088525)
Supplement: Supplementary file 1 [file Table1.docx]

**Supplementary Table Comparison of** **the laboratory findings of KD-MAS patients before and after the first dose of IVIG treatment**

| Laboratory findings | Before treatment | After treatment | *Z/t*-test | *P* |
| --- | --- | --- | --- | --- |
| WBC [×10^9/L, (*P_25_, P_75_*)] | 10.45(5.22,16.65) | 12.64(7.54,21.40) | -1.065 | 0.287 |
| Neutrophils count [×10^9/L, (*P_25_, P_75_*)] | 7.19(2.67,11.10) | 8.32(2.13,10.55) | -0.361 | 0.718 |
| NLR [M (*P_25_, P_75_*)] | 7.19(2.67,11.10) | 1.83(0.74,3.72) | -1.090 | 0.276 |
| PLT [×10^9/L, M (*P_25_, P_75_*)] | 69.00(48.50,177.25) | 122.50(95.00,163.75) | -1.975 | 0.048 |
| Hb (g/L,$\bar{x}\pm s$) | 102.00±10.97 | 92.43±13.49 | 2.913 | 0.005 |
| Hs-CRP [mg/L, M (*P_25_, P_75_*)] | 105.50(61.00,156.00) | 31.45(13.63,56.43) | -3.515 | 0.000 |
| PCT [mg/L, M (*P_25_, P_75_*)] | 1.54(0.58,3.08) | 0.84(0.58,1.30) | -2.172 | 0.030 |
| CL^-^ [mmol/L, M (*P_25_, P_75_*)] | 99.35(96.85,100.90) | 101.25(98.40,102.60) | -1.943 | 0.052 |
| Na^+^ (mmol/L,$\bar{x}\pm s$) | 135.73±4.05 | 137.33±5.04 | -1.309 | 0.196 |
| TG [mmol/L, M (*P_25_, P_75_*)] | 2.43(1.79,3.58) | 3.14(2.56,3.94) | -1.983 | 0.047 |
| Ferritin [ng/mL, M (*P_25_, P_75_*)] | 935.66(655.50,2035.85) | 1821.44(1191.65,6003.78) | -2.417 | 0.016 |
| FIB [g/L, M (*P_25_, P_75_*)] | 1.65(133.53,139.15) | 1.77(1.52,2.34) | -0.885 | 0.376 |
| Albumin [g/L, M (*P_25_, P_75_*)] | 28.90(24.15,35.48) | 28.05(25.53,29.20) | -1.246 | 0.213 |
| ALT [U/L, M (*P_25_, P_75_*)] | 94.50(51.25,130.50) | 46.00(29.25,132.00) | -1.336 | 0.182 |
| AST [U/L, M (*P_25_, P_75_*)] | 78.00(47.00,140.25) | 106.00(34.00,195.00) | -0.566 | 0.572 |
| LDH [U/L, M (*P_25_, P_75_*)] | 450.50(336.50,847.00) | 373.00(299.25,562.75) | -1.295 | 0.195 |

**KD, Kawasaki disease; MAS, macrophage activation syndrome; IVIG, intravenous immunoglobulin; WBC, white blood cell count; NLR, neutrophil-to-lymphocyte count ratio; PLT, platelet; Hb, hemoglobin; Hs-CRP, hypersensitive C-reactive protein; PCT, procalcitonin; TG, triglyceride; FIB, Fibrinogen; ALT, alanine aminotransferase; AST, aspartate aminotransferase; LDH, lactic dehydrogenase.**


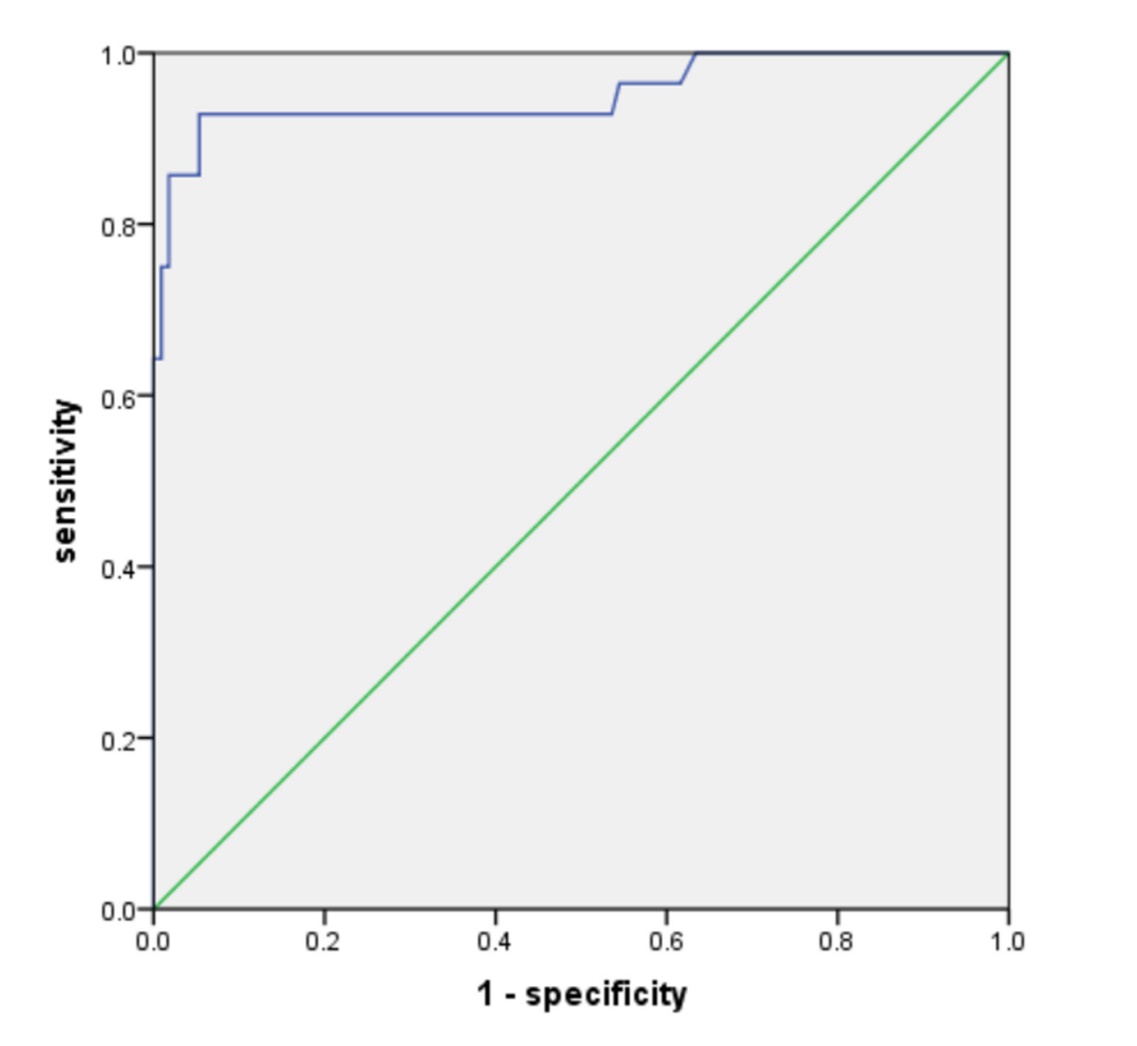


**Supplementary Figure 1 Receiver operating characteristic (ROC) curve analysis of the final model in predicting the development of mas in children with KD.**


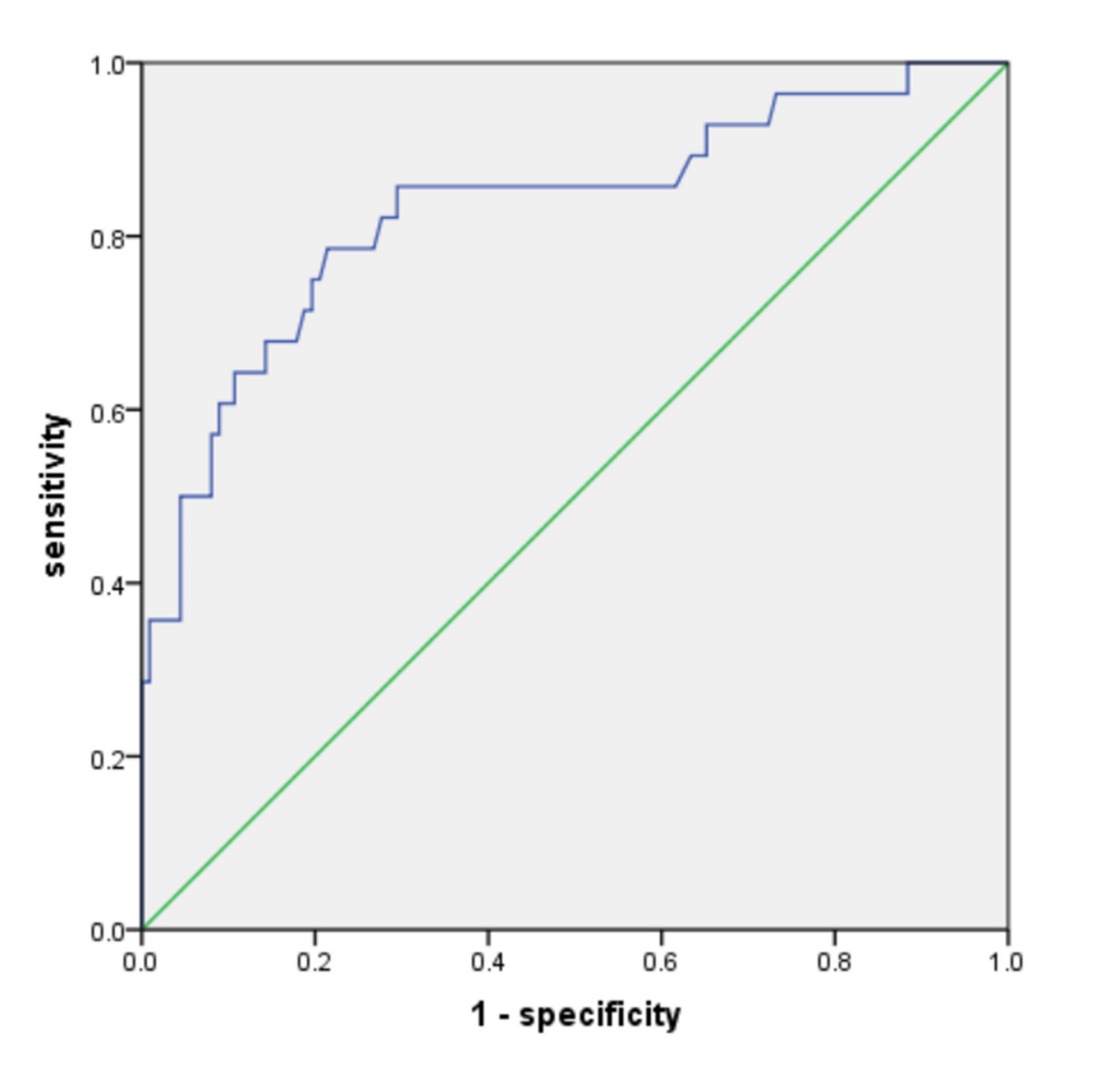


**Supplementary Figure 2 Receiver operating characteristic (ROC) curve analysis of the final model by leave-one-out cross-validation in predicting the development of mas in children with KD.**
